# Nonadiabatic reaction dynamics to silicon monosulfide (SiS): A key molecular building block to sulfur-rich interstellar grains

**DOI:** 10.1126/sciadv.abg7003

**Published:** 2021-06-25

**Authors:** Srinivas Doddipatla, Chao He, Shane J. Goettl, Ralf I. Kaiser, Breno R. L. Galvão, Tom J. Millar

**Affiliations:** 1 Department of Chemistry, University of Hawai‘i at Mānoa, Honolulu, HI 96822, USA.; 2 Centro Federal de Educação Tecnológica de Minas Gerais, Av. Amazonas 5253, 30421-169 Belo Horizonte, Brazil.; 3 School of Mathematics and Physics, Queen’s University Belfast, Belfast BT7 1NN, Northern Ireland, UK.

## Abstract

Sulfur- and silicon-containing molecules are omnipresent in interstellar and circumstellar environments, but their elementary formation mechanisms have been obscure. These routes are of vital significance in starting a chain of chemical reactions ultimately forming (organo) sulfur molecules—among them precursors to sulfur-bearing amino acids and grains. Here, we expose via laboratory experiments, computations, and astrochemical modeling that the silicon-sulfur chemistry can be initiated through the gas-phase reaction of atomic silicon with hydrogen sulfide leading to silicon monosulfide (SiS) via nonadiabatic reaction dynamics. The facile pathway to the simplest silicon and sulfur diatomic provides compelling evidence for the origin of silicon monosulfide in star-forming regions and aids our understanding of the nonadiabatic reaction dynamics, which control the outcome of the gas-phase formation in deep space, thus expanding our view about the life cycle of sulfur in the galaxy.

## Teaser

This study reveals the nonadiabatic dynamics of silicon monosulfide and its role in the formation of sulfur-rich grains.

## INTRODUCTION

For more than half a century, the origin of interstellar grains—nanoparticles with refractory sulfide (*1*), silicate (*2*), silicon carbide (*3*), and/or carbonaceous cores (*4*)—has remained a controversial topic because interstellar grains are thought to be faster destroyed by interstellar shocks than formed during the late stages of stellar evolution through nucleation in supernova remnants and in the circumstellar envelopes of asymptotic giant branch (AGB) and red supergiant stars (*1*, *5*–*11*). Thus, the omnipresence of grains in the interstellar medium (ISM) signifies one of the most decisive enigmas in astrophysics (*12*, *13*). This is emphasized by the discrepancy between the formation rates of grains in circumstellar envelopes of a few 10^9^ years and their destruction, once injected into the ISM, constraining their lifetime to only some 10^8^ years (*8*, *14*–*16*). This inconsistency might be ultimately answered through a better understanding of the fundamental molecular mass growth processes and the knowledge of molecular precursors associated with the formation of these nanostructures on the most fundamental, microscopic level (*17*–*20*). In deep space, nanoparticles play a critical role in star formation and in the origin of solar systems by controlling the thermal balance (*2*, *3*) and in promoting catalytic surface reactions (*2*, *4*). Interstellar grains have also been connected to the prebiotic evolution through the formation of molecular building blocks of life such as amino acids and sugars by energetic processing (*21*) on their ice-coated surfaces. Consequently, the elucidation of the origin and growth of nanoparticles is vital to eventually understand the fundamental processes that create a visible galaxy including our own.

**Fig. 1:**
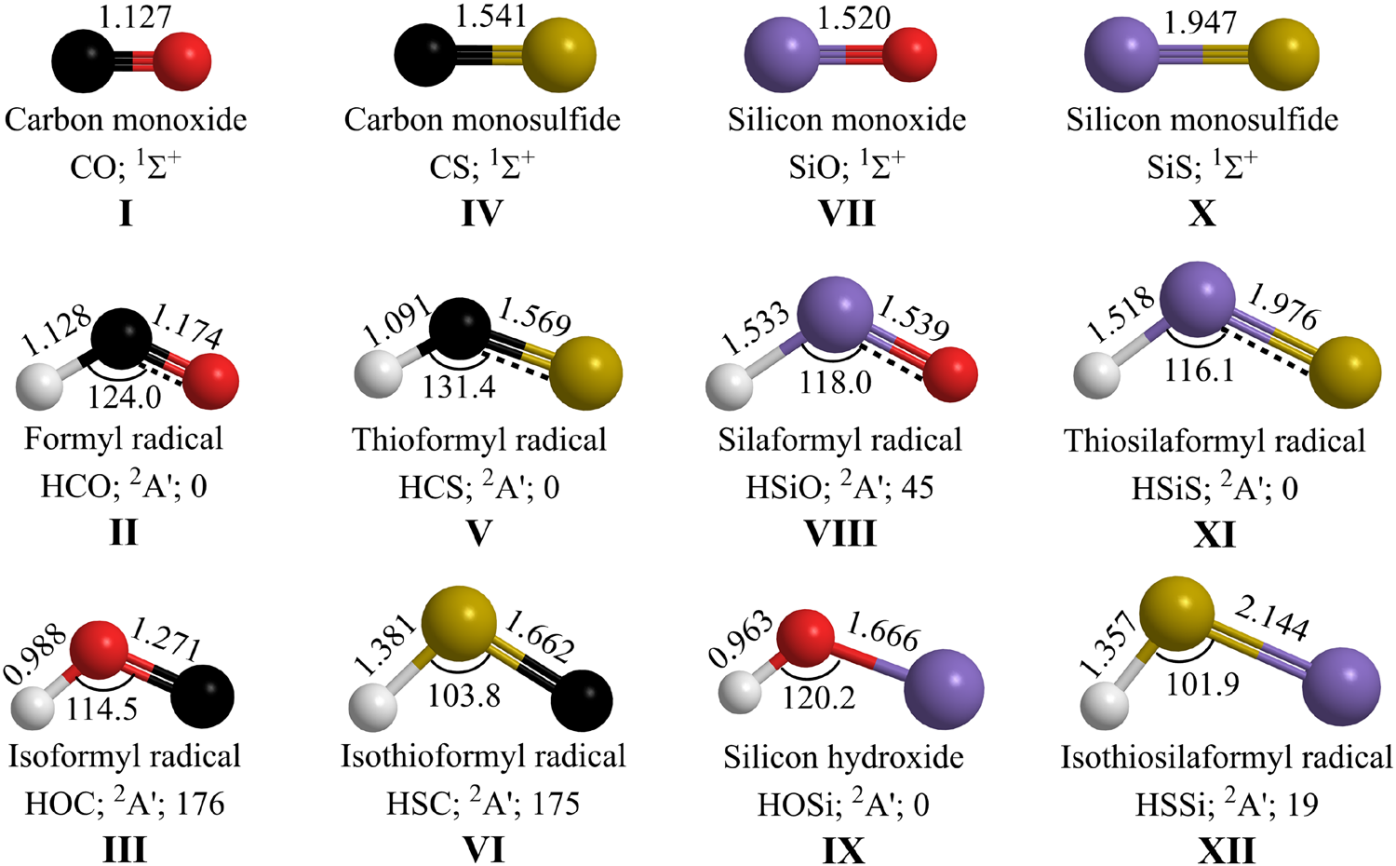
Geometries of HXY (*X* = C, Si; *Y* = O, S) isomers and corresponding diatomic (*XY*) molecules. Relative energy of the triatomic molecules with respect to the most stable isomer is given in kilojoule per mole. Bond lengths are in angstroms (Å), and angles in degrees (°). Colors of the atoms: carbon (black), oxygen (red), sulfur (yellow), silicon (purple), and hydrogen (white).

Whereas an understanding of the formation of carbonaceous and silicate-based grains through the involvement of polycyclic aromatic hydrocarbons (PAHs) (*22*) and silicon oxides (*23*, *24*) is beginning to emerge, the knowledge of the elementary processes leading to sulfide grains is in its infancy. Astronomical observations (*25*–*29*) along with astrochemical modeling (*30*–*34*) advocate that silicon monosulfide [SiS(X^1^Σ^+^)] represents the critical molecular building block initiating the chain of reactions that lead ultimately to sulfide dust grains (Fig. 1). Silicon monosulfide has been detected in circumstellar envelopes of carbon- and oxygen-rich AGB stars with mean fractional gas-phase abundances of 3.1 × 10^−6^ and 2.7 × 10^−7^, respectively (*27*), in chemically active regions, where dust grains are formed and mass loss from the central star exceeds rates of 10^−6^ solar masses per year (*11*). The 6.6- and 13.5-μm emission bands around S-type AGB stars (C/O ≈ 1) have been attributed to silicon monosulfide (*33*). This molecule was also detected toward star-forming regions such as SgrB2 (*29*), Orion KL (*28*), and L1157-B1 (*13*), with evidence of shock waves in the molecular outflows in the latter source, suggesting that silicon monosulfide is either ablated from the refractory grain or formed in the postshock zone through coupling of the silicon and sulfur chemistries. Astrochemical models postulate the formation of silicon monosulfide is linked to reactions of interstellar anions with the silicon monosulfide cation (SiS^+^), proton transfer from protonated silicon monosulfide (HSiS^+^), and dissociative recombination of protonated silicon monosulfide with an electron (*35*). Alternatively, radiative associations between atomic silicon and sulfur (*36*) along with bimolecular collisions of atomic silicon with the thioxydroxyl radical (SH) (*37*) and with sulfur monoxide (SO) (*38*, *39*) have been assumed to initiate the formation of the first silicon-sulfur bond. Considering that none of the aforementioned postulated pathways to silicon monosulfide have been explored in laboratory studies, it is not unexpected that astrochemical models underestimate the astronomical observed fractional abundances of silicon monosulfide by up to two orders of magnitude (*30*). Therefore, the underlying formation pathways of the fundamental molecular building block to sulfide grains—the diatomic silicon monosulfide molecule—are still elusive. An understanding of the synthetic routes to silicon monosulfide as a tracer of sulfide grain formation is critical not only to provide constraints on the role of sulfide dust condensation in regulating the chemistry in circumstellar envelopes but also to explore the unknown pathways leading to the very first silicon-sulfur bond on the molecular level and to objectively identify the key molecular species contributing to dust formation.

Here, we reveal through crossed molecular beam experiments and electronic structure calculations that the silicon monosulfide molecule (SiS) along with D1-thiosilaformyl radical (DSiS) can be efficiently formed via the elementary reaction of ground-state atomic silicon [Si(^3^P_j_)] with deuterium sulfide (D_2_S) in the gas phase involving nonadiabatic reaction dynamics via rovibrationally excited [SiD_2_S]* intermediate(s) (reactions 1 and 2). This system signifies a prototype of a reaction of ground-state atomic silicon generated via the photolysis and cosmic ray decomposition of silane (SiH_4_) (*24*, *40*, *41*) with hydrogen (deuterium) sulfide—the parent species of the interstellar sulfur chemistry (Materials and Methods). In star-forming regions, where gas-phase chemistry follows ice mantle sublimation, and in circumstellar envelopes of high-mass loss stars with sufficient fractional abundances of hydrogen sulfide (*42*, *43*), the reaction of atomic silicon with hydrogen sulfide may represent a potential pathway to form silicon monosulfide in a single collision event. Silicon monosulfide may then drive an exoergic chemistry (*27*, *32*) that produces ultimately sulfide grains via fundamental molecular mass growth processes, bringing us closer to understanding the life cycle of interstellar dust in our universe

Si(Pj 3)+D2S (X1A1)→[SiD2S]*→DSiS (X2A′)+D (S 2) (1)

Si(Pj 3)+D2S (X1A1)→[SiD2S]*→SiS (X1Σ+)+D2(X1Σg+) (2)

## RESULTS

### Crossed molecular beam studies—Laboratory frame

**Fig. 2:**
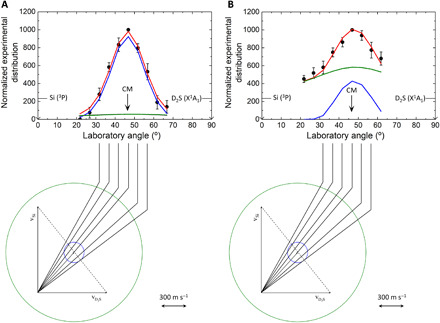
Newton diagrams and laboratory angular distributions of products for the reaction of Si(^3^P) + D_2_S(X^1^A_1_). Lower: Newton diagrams for the reaction Si(^3^P) + D_2_S(X^1^A_1_) at collision energy of 12.7 kJ mol^−1^. The circles stand for the maximum center-of-mass recoil velocity of molecular (green) and atomic (blue) deuterium loss channels leading to, from outer to inner, silicon monosulfide (SiS) and D1-thiosilaformyl radical (SSiD). Upper: Laboratory angular distributions of the products recorded at *m/z* = 62 (^28^Si^32^SD^+^/^28^Si^34^S^+^/^30^Si^32^S^+^). ^28^Si^32^SD^+^ originates from the reactions of ground-state atomic silicon [^28^Si(^3^P)] with deuterium sulfide (D_2_^32^S; X^1^A_1_) (blue) via deuterium atom loss. ^28^Si^34^S^+^/^30^Si^32^S^+^ originate from the reactions of ground-state atomic silicon [^28^Si/^30^Si (^3^P)] with deuterium sulfide (D_2_^34^S/ D_2_^32^S; X^1^A_1_) via molecular deuterium loss (green/purple) (**A**). Laboratory angular distributions of the products recorded at *m/z* = 60 (SiS^+^). The fits in green and blue color represent the molecular deuterium loss channel and the dissociative ionization of SSiD formed via atomic deuterium loss, respectively (**B**). The solid circles represent the experimental data, CM designates the center-of-mass angle, the error bars represent the 1σ SD, and red solid lines represent the overall fit. The solid lines point to distinct laboratory angles whose TOFs are shown in Fig. 3.

**Fig. 3:**
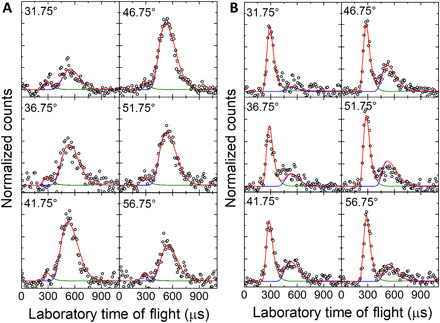
Time-of-flight data. TOF data at *m/z* = 62 (**A**) and *m/z* = 60 (**B**) for laboratory angle 31.75°, 36.75°, 41.75°, 46.75°, 51.75°, and 56.75° at a collision energy 12.7 kJ mol^−1^. Open circles depict the experimental data, and solid line the fit.

The crossed molecular beam experiments were performed by intersecting supersonic beams of ground-state atomic silicon (Si; ^3^P) with deuterium sulfide (D_2_S; X^1^A_1_) perpendicularly in gas phase under single-collision conditions at collision energy of 12.7 ± 0.4 kJ mol^−1^ (Materials and Methods; table S1). Neutral reaction products were ionized via electron impact within a triply differentially pumped quadrupole mass spectrometer followed by mass selection and collection of time-of-flight (TOF) spectra at distinct laboratory angles. Reactive scattering signal was collected at mass-to-charge ratios (*m/z*) from 64 to 60 to probe potential adducts [^28^SiD_2_^32^S, 64 atomic mass units (amu)] along with the atomic and molecular deuterium loss channels leading to the D1-(iso)thiosilaformyl radical (D^28^Si^32^S/ D^32^S^28^Si) (62 amu) and silicon monosulfide (^28^Si^32^S) (60 amu) (reactions 1 and 2). Reactive scattering signal was observed at *m/z* = 62 (^28^Si^32^SD^+^/^28^Si^34^S^+^/^30^Si^32^S^+^/^29^Si^33^S^+^) and *m/z* = 60 (^28^Si^32^S^+^) (Figs. 2 and 3). At each angle, the TOF spectra at *m/z* = 62 and 60 are distinct and do not overlap after scaling, suggesting that multiple channels are open in this elementary reaction. As shown in Fig. 3, the TOF spectra at *m/z* = 62 exhibit two distinct peaks revealing maxima close to 300 and 550 μs with an intensity ratio of, e.g., 0.03 ± 0.01 at the center-of-mass (CM) angle of 46.3° ± 0.8°. The TOF spectra at *m/z* = 60 also expose a bimodal pattern with maxima near to 300 and 550 μs, albeit with an inverted intensity ratio at the CM angle of 46.3° ± 0.8° of 1.3 ± 0.4. The corresponding laboratory angular distributions (LAD), which report the integrated and scaled signal of *m/z* = 62 and *m/z* = 60 of the TOF spectra versus the laboratory angles, are also distinct, with scattering signal for *m/z* = 62 spread over at least 46° in the scattering plane (Fig. 2). The laboratory angular distribution of *m/z* = 60 reveals a much broader distribution, which does not overlap with the data at *m/z* = 62; this finding suggests that signal at *m/z* = 60 does not (only) arise from dissociative electron impact ionization of products with a molecular mass of 62 amu. Signal at *m/z* = 61, 63, and 64 may arise from the several combinations of silicon and sulfur isotopes, but their contributions were too weak to be monitored; this is in agreement with the low natural abundance of isotopes of silicon and sulfur (table S2). An inspection of the corresponding Newton diagrams for the atomic and molecular deuterium loss channels (reactions 1 and 2, respectively) reveals interesting findings (Fig. 2). These diagrams correspond to the most probable velocities of the atomic silicon and deuterium sulfide reactants; the two-dimensional projection of the recoil spheres (“Newton circles”) indicates the maximum velocities of the recoil vectors for channels 1 and 2, accounting for conservation of energy and linear momentum (*44*). Considering the “expected” angular range of the D^28^Si^32^S/D^32^S^28^Si (62 amu) and ^28^Si^32^S (60 amu) products, the Newton circles for the atomic and molecular deuterium loss channels are quite distinct with the angular range of signal collected at *m/z* = 62, revealing close resemblance to the theoretically expected distribution of the atomic deuterium loss channel (reaction 1). The predicted angular range of the molecular deuterium loss pathway (reaction 2) is significantly larger than the Newton circle of the atomic deuterium loss and could account for the signal at *m/z* = 60. Therefore, our laboratory data may suggest the existence of the atomic and molecular deuterium loss leading to the D1-(iso)thiosilaformyl radical (D^28^Si^32^S) and silicon monosulfide (^28^Si^32^S), respectively.

### Crossed molecular beams studies—CM frame

**Fig. 4:**
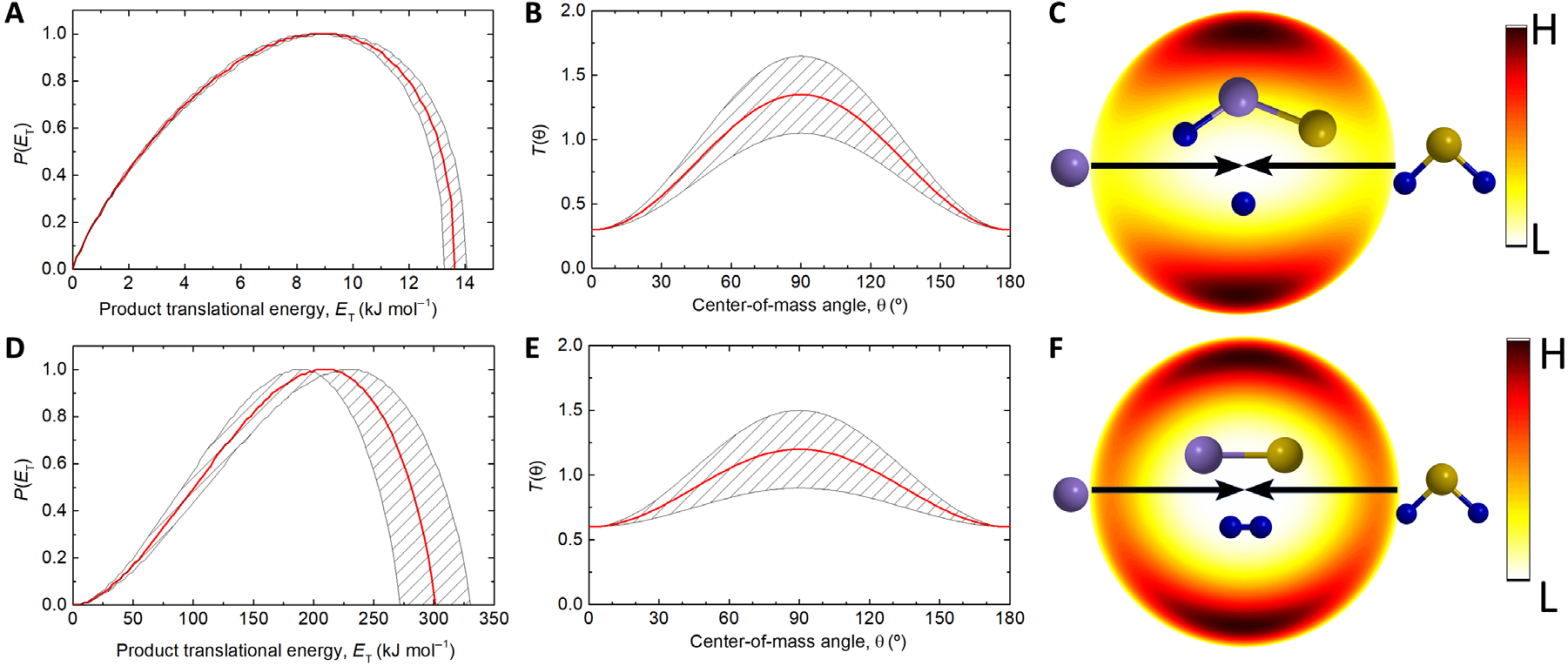
Center-of-mass functions. Center-of-mass translational energy *P*(*E*_T_) (**A** and **D**) and angular *T*(θ) (**B** and **E**) flux distributions for the reaction of ground state atomic silicon [Si(^3^P)] with deuterium sulfide (D_2_S; X^1^A_1_). (A) and (B) are responsible for the formation of SSiD plus atomic deuterium products. (D) and (E) depict the formation of SiS plus molecular deuterium. Flux contour map for the formation of D1-thiosilaformyl radical (DSiS; ^2^A′) (**C**) and silicon monosulfide (SiS; X^1^∑^+^) (**F**).

To provide quantitative evidence on the existence of the atomic and molecular deuterium loss channels in the reaction of ground-state atomic silicon with deuterium sulfide and to acquire information on the chemical dynamics, we are transforming now the experimental data (LAD, TOF) from the laboratory to the CM reference frame (*45*, *46*). This approach eventually results in the CM translational energy flux distribution *P*(*E*_T_) and the CM angular flux distribution *T*(θ) (Fig. 4). First, best fits of the slow, more intense peak of the TOF spectra of *m/z* = 62 could be achieved with a single-channel fit of the reaction of silicon (^28^Si; 28 amu) with deuterium sulfide (D_2_^32^S; 36 amu), yielding products with a mass combination of 62 amu (D^28^Si^32^S) and 2 amu (D). These sections of the TOFs of *m/z* = 62 overlap with the less intense peaks of the TOF spectra collected at *m/z* = 60 after scaling, suggesting that signal at *m/z* = 60 in the range of 450 to 800 μs arises from dissociative electron impact ionization of the neutral D^28^Si^32^S product isomer(s) detectable as ^28^Si^32^S^+^ at *m/z* = 60. Second, signal at the fast parts of the TOFs at *m/z* = 60 could be replicated with the molecular deuterium loss channel forming the heavy silicon monosulfide cofragment (^28^Si^32^S). The fast peaks at *m/z* = 60 reveal—after scaling—identical pattern to the fast section of the TOFs recorded at *m/z* = 62. These sections of the TOFs at *m/z* = 62 could be fit via the reactions of isotopically substituted counterparts of the reactants (^28^Si/D_2_^34^S; ^30^Si/D_2_^32^S), leading to ^28^Si^34^S/^30^Si^32^S (62 amu) along with molecular deuterium. The intensity ratio of the fast peaks at *m/z* = 60 to *m/z* = 62 of 25 ± 7 is well replicated by the intensity ratios of naturally occurring isotopes of silicon and sulfur, i.e., ^28^Si/^30^Si = 30 and ^32^S/^34^S = 22. To sum up, the reactive scattering data propose the existence of the atomic and molecular deuterium loss channels leading to D1-(iso) thiosilaformyl radical (D^28^Si^32^S/D^32^S^28^Si; hereafter DSiS/DSSi) (62 amu) and silicon monosulfide (^28^Si^32^S; hereafter SiS) (60 amu).

In detail, fits could be achieved with CM translational energy distributions for the atomic and molecular deuterium loss channels exhibited in Fig. 4 (A and D) extended up to a maximum translational energy (*E*_max_) of 13.5 ± 0.4 kJ mol^−1^ (D loss) and 301 ± 29 kJ mol^−1^ (D_2_ loss), respectively. Energy conservation commands that for reaction products formed without internal excitation, the high energy cutoff denotes the sum of the reaction exoergicity and the collision energy *E*_c_ (12.7 ± 0.4 kJ mol^−1^). Hence, a subtraction of the collision energy reveals that the reactions 1 and 2 are weakly and strongly exoergic by 0.8 ± 0.4 kJ mol^−1^ (DSiS/DSSi) and 288 ± 29 kJ mol^−1^ (SiS), respectively. Further, both *P*(*E*_T_)s peak away from zero translational energy at 9 and 200 kJ mol^−1^ for both channels, suggesting tight exit transition states of the decomposing reaction D_2_SiS intermediates along with a substantial rearrangement of the electron density to form silicon monosulfide along with molecular deuterium. Last, both *T*(θ)s are forward-backward symmetric and disclose intensity from 0° to 180°. These findings propose indirect scattering dynamics involving long-lived D_2_SiS intermediate(s) with lifetimes longer than or at least comparative with their rotation periods. The distribution maxima at 90° hint geometrical limitations with the atomic and molecular deuterium ejected nearly parallel to the total angular momentum vector nearly perpendicular to the rotational plane of the fragmenting D_2_SiS complex(es) (*47*, *48*). These findings are also compiled in the flux contour maps (Fig. 4) for the atomic and molecular deuterium loss channels revealing fractions of 5 ± 1 and 95 ± 3%, respectively (*49*).

### Electronic structure calculations—Reaction products

**Fig. 5:**
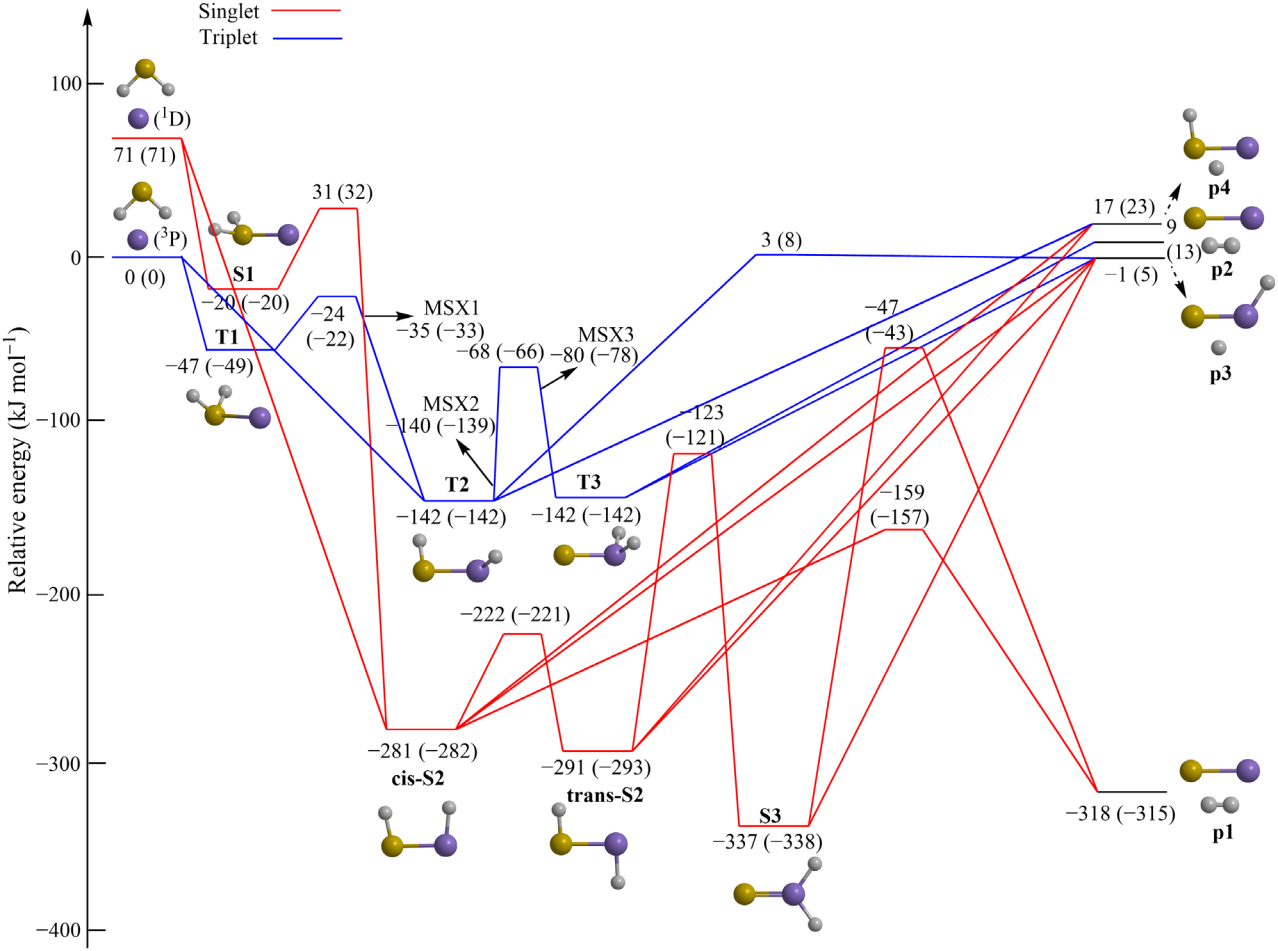
PES for the reactions of hydrogen sulfide (H_2_S) with atomic silicon (Si) at the MRCI-F12/cc-pVQZ-F12//CASSCF/aug-cc-pV(Q + d)Z level. Relative energies are given in units of kilojoule per mole. Plain numbers are zero point energies corrected for hydrogenated reactants, whereas numbers in parentheses are for deuterated reactants. Colors of the atoms are defined as sulfur (yellow), silicon (purple), and hydrogen (gray). The complete PES including Cartesian coordinates and vibrational frequencies along with transition states not accessible under our experimental conditions are compiled in fig. S1 and table S3.

Having identified the atomic and molecular deuterium loss channels forming DSiS/DSSi and SiS, respectively, as the product of the bimolecular gas-phase reaction of ground-state silicon atoms with deuterium sulfide (reactions 1 and 2), we are elucidating now the underlying reaction mechanism(s) and chemical dynamics. This is accomplished by merging our experimental findings with accurate electronic structure calculations on the triplet and singlet D_2_SiS potential energy surfaces (PESs). The structures of the reactants, products, intermediates, transition states, and singlet-triplet seam of crossings [minima on the seam of crossings (MSX)] connected to the reaction of ground-state atomic silicon (Si; ^3^P) with deuterium sulfide (D_2_S; X^1^A_1_) were optimized with the full-valence complete-active-space-self-consistent-field (CASSCF)/aug-cc-pV(Q + d)Z method (*50*–*52*), and vibrational frequencies were calculated at the same level. The energy values were refined using the explicitly correlated multireference configuration interaction (*53*–*55*) (MRCI-F12) method with the cc-pVQZ-F12 basis set (*56*) [MRCI-F12/cc-pVQZ-F12//CASSCF/aug-cc-pV(Q + d)Z]. This method is known to achieve chemical accuracy (*53*) within ±8 kJ mol^−1^. The results are assembled in Fig. 5 and fig. S1. These calculations predict the existence of four exit channels to products **p1** to **p4**. The atomic deuterium loss to form the D1-thiosilaformyl radical (DSiS; X^2^A′, **p3**) is slightly endoergic by 5 kJ mol^−1^ or within the error limits slightly exoergic with respect to the separated reactants; the D1-isothiosilaformyl isomer (DSSi; X^2^A′; **p4**) is thermodynamically less stable than **p3** by 18 kJ mol^−1^ and, hence, cannot be accessed in our experiment considering a collision energy of 12.7 ± 0.4 kJ mol^−1^. The computed energy difference between the C_s_ symmetric isomers **p3** and **p4** of 18 kJ mol^−1^ is in excellent agreement with results from Rosi *et al.* (*39*), Pérez-Justea and Carballeira (*57*), and Bruna and Grein (*58*), favoring **p3** by 15 to 19 kJ mol^−1^. In the D1-thiosilaformyl radical (DSiS; X^2^A′, **p3**), the deuterium atom is bonded to the silicon atom, creating a strong σ_Si-D_ bond, whereas silicon and sulfur favor an efficient overlap of their pπ orbitals with a bond distance of 195.7 pm ranging between a double and triple bond; in DSSi (X^2^A′, **p4**), the silicon-sulfur bond is weaker and can be characterized as a double bond (212.3 pm) (Supplementary Materials). The experimentally determined reaction energy of −0.8 ± 0.4 kJ mol^−1^ correlates well with the computed value of +5 ± 8 kJ mol^−1^ to form the D1-thiosilaformyl radical (DSiS; X^2^A′, **p3**). Considering the molecular deuterium loss, the formation of ground-state silicon monosulfide (SiS; X^1^∑^+^, **p1**) is computed to be strongly exoergic by 315 ± 8 kJ mol^−1^ on the singlet surface. This result correlates nicely with the experimentally derived exoergicity of −288 ± 29 kJ mol^−1^. On the triplet surface, triplet silicon monosulfide (**p2**) could be formed in its a^3^Π state in an overall endoergic reaction (13 ± 8 kJ mol^−1^). Accounting for our collision energy of 12.7 ± 0.4 kJ mol^−1^ and the *P*(*E_T_*) of the molecular deuterium loss channel, upper limits, if any, of less than 1% of **p2** can be derived. Therefore, these data reveal the existence of the formation of the D1-thiosilaformyl radical (DSiS; X^2^A′, **p3**) and silicon monosulfide (SiS; X^1^∑^+^, **p1**) with possibly minor contributions from triplet silicon monosulfide (SiS; a^3^Π^+^, **p2**).

### Electronic structure calculations—Triplet surface

The computations reveal also the existence of three reaction intermediates on the triplet surface. The reaction of atomic silicon (Si; ^3^P) with deuterium sulfide (D_2_S; X^1^A_1_) was found to be initiated barrierlessly on the triplet surface via addition of silicon to one of the nonbonding electron pairs of sulfur, yielding a collision complex **T1** (D_2_SSi, **T1**, C_s_, ^3^A″) or through insertion of atomic silicon into one of the sulfur—deuterium bonds forming D1-thiohydroxysilylene (DSSiD; **T2**, C_1_, ^3^A). **T1** and **T2** are connected via a barrier of only 27 kJ mol^−1^ with respect to **T1** through deuterium atom migration. A deuterium shift connects **T2** to D2-silathioformaldehyde (D_2_SiS; **T3**, C_s_, ^3^A″) by passing a transition state located 76 kJ mol^−1^ above **T2**. Both **T2** and **T3** can undergo unimolecular decomposition to the D1-thiosilaformyl radical (DSiS; X^2^A′, **p3**). Considering the singlet multiplicities of the silicon monosulfide (SiS; X^1^∑^+^) and molecular deuterium (D_2_; X^1^∑_g_^+^) products, as well as the triplet and singlet multiplicities of the ground-state silicon and deuterium sulfide reactants, nonadiabatic reaction dynamics and intersystem crossing (ISC) from the triplet to the singlet manifold must play a critical role in the formation of silicon monosulfide (SiS; X^1^∑^+^), and an investigation of the singlet manifold is also warranted.

### Electronic structure calculations—Singlet surface

On the singlet surface, the calculations identified four intermediates. There, **S1 (**D_2_SSi, **S1**, C_s_, ^1^A′) may undergo a deuterium shift to cis-D2-thiohydroxysilylene (DSSiD; **cis-S2**, C_s_, ^1^A′). Since the inherent barrier of the deuterium shift of 32 kJ mol^−1^ is higher than the collision energy, under our experimental study, this isomerization process is closed energetically. An isomerization links **cis-S2** via trans-D2-thiohydroxysilylene (DSSiD; **trans-S2**, C_s_, ^1^A′) to D_2_SiS (**S3**, C_2v_, ^1^A_1_)—the global minimum of the D_2_SiS PES. Owing to the repulsion of the Si-D and S-D sigma bonds, **cis-S2** is destabilized by 11 kJ mol^−1^ with respect to **trans-S2**. The relative stabilities of these isomers are in good agreement with previous results of 10 to 11 kJ mol^−1^ (**cis-S2** versus **trans-S2**) and 39 to 45 kJ mol^−1^ (**trans-S2** versus **S3**) (*59*–*61*). Three intermediates (**cis-S2**, **trans-S2**, and **S3**) may undergo a barrierless atomic deuterium loss to the D1-thiosilaformyl radical (DSiS; X^2^A′, **p3**) via simple bond rupture processes; both **cis-S2** and **S3** can emit molecular deuterium via tight transition states located 157 and 43 kJ mol^−1^ below the energy of the separated reactants leading to silicon monosulfide (SiS; X^1^∑^+^).

### Electronic structure calculations—ISC

To evaluate the possibility of ISC from the triplet to singlet surface, minima-on-the-seam-of-crossings (MSX) were explored. These investigations identified three minima-on-the-seam-of-crossings (**MSX1** to **MSX3**). **MSX1** is located in vicinity of the transition state connecting **T1** and **T2** and connects to **cis**-**S2** with a spin-orbit coupling (SOC) of 71 cm^−1^. **MSX2** lies close to **T2** both in energy and geometry early in the isomerization to **T3** and connects to **S3**. **MSX3** is also connected to the **T2**→**T3** isomerization, but it is located late in the reaction coordinate ranging 61 kJ mol^−1^ higher in energy than **MSX2**, leading to **S3**. **MSX2** and **MSX3** are linked to SOCs of 46 and 29 cm^−1^, respectively, substantially lower than **MSX1**. Note that Fig. 5 symbolizes a simplified, one-dimensional representation of a six-dimensional PES; this constrains the graphical representation of all **MSXs**, since the surface can only visualize minimum energy paths between the intermediates; thus, each **MSX** is represented with only the energy.

### Reaction mechanisms and chemical dynamics

**Fig. 6:**
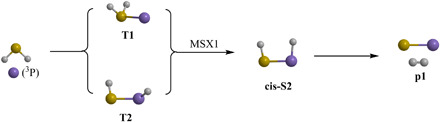
Favorable reaction mechanisms leading to silicon monosulfide (SiS) via nonadiabatic reaction dynamics.

The electronic structure calculations and experimental data reveal that the reaction of ground-state atomic silicon with D2-hydrogen sulfide proceeds via indirect scattering dynamics involving long-lived SiD_2_S reaction intermediates and is initiated by the barrierless addition of silicon to one of the nonbonding electron pairs at the sulfur atom forming intermediate **T1** and possibly via insertion into one of both sulfur-deuterium single bonds leading to **T2** (Fig. 6). Considering the larger cone of acceptance of the addition process compared with the geometrically constrained insertion, silicon-sulfur coupling to **T1** is likely favorable compared with **T2**. Both intermediates are connected via a transition state; this isomerization process passes the minima-on-the-seam-of-crossing **MSX1** and facilitates ISC from the triplet to the singlet surface to **cis-S2**. This intermediate can undergo molecular deuterium loss to form silicon monosulfide (**p1**) in an overall exoergic reaction (experiments: −288 ± 29 kJ mol^−1^; theoretical: −315 ± 8 kJ mol^−1^) via a tight exit transition state located 157 kJ mol^−1^ above the separated products. The computed geometry of the exit transition state suggests that this molecular deuterium loss proceeds nearly parallel to the total angular momentum vector at an angle of only 5.3° (fig. S3) with the tight exit transition state along with the geometrical constraints predicted from the CM translational and angular distributions (Fig. 4). Note that an alternative molecular deuterium loss pathway was identified computationally to proceed via **S3**, which, in principle, is accessible via the isomerization sequence **cis-S2**→**trans**-**S2**→**S3**. However, although the exit transition state is also tight, the computed geometry of the exit transition state predicts a molecular deuterium loss within the rotational plane of the decomposing complex nearly perpendicularly to the total angular momentum, which does not correlate with the aforementioned experimental findings of a molecular deuterium loss parallel to the angular momentum vector. Therefore, we can conclude that, most likely, intermediate **S3** does not play a role in the formation of silicon monosulfide (**p1**) and that the preparation of the latter plus molecular deuterium has to proceed via nonadiabatic reaction dynamics involving the sequence **T1/T2**→**MSX1**→**cis-S2**→**p1 + D**_**2**_. This conclusion is also in line with the favorable energies of the transition states in the aforementioned reaction sequence compared with **T1/T2**→**MSX1**→**cis-S2**→**trans**-**S2**→**S3**→**p1 + D**_**2**_ and/or the favorable SOC of 71 cm^−1^ for **MSX1** compared with 46 and 29 cm^−1^ for **MSX2** and **MSX3**, respectively, if **S3** is accessed via those minima-on-the-seam-of-crossings. Last, the atomic deuterium loss channel to form the D1-thiosilaformyl radical (DSiS; X^2^A′, **p3**) only accounts for up to 5% of the scattering signal, with the laboratory data and electronic structure calculations predicting indirect scattering dynamics as well. On the triplet surface, unimolecular decomposition of **T3** could yield the D1-thiosilaformyl radical without exit barrier; on the singlet surface, **cis-S2, trans**-**S2,** and/or **S3** may yield the D1-thiosilaformyl radical.

### Astrochemical modeling

Astrochemical modeling is essential since molecular beam experiments conducted under well-defined laboratory conditions and computations hardly simulate the chemical complexity in star-forming regions. For technical reasons, the molecular beam experiments had to be conducted with deuterium sulfide (D_2_S) (Materials and Methods), whereas in deep space, hydrogen sulfide (H_2_S) prevails. However, electronic structure calculations reveal that accounting for the zero point energies of the reactants and products, the overall reaction exoergicities change only slightly from 315 to 318 kJ mol^−1^ (Fig. 5). Likewise, these computations demonstrate that the reaction has no entrance barrier; all barriers to isomerization are well below the energy of the separated reactants. Significant entrance barriers would prohibit these reactions in low-temperature environments. Therefore, our results can be applied to those star-forming regions, in which ice mantles, and most importantly hydrogen sulfide, can be removed from grains by sublimation (*62*), and where adequate concentrations of silicon atoms exist along with hydrogen sulfide.

Our crossed-beam, single-collision study of reaction between silicon atoms and hydrogen sulfide together with our electronic structure calculations allow us to make a quantitative assessment of the potential of this reaction to form silicon monosulfide (SiS; X^1^∑^+^, **p1**) in space. We conduct this through astrochemical kinetic modeling of three regions in the Orion Kleinmann-Low nebula, the closest region of massive star formation to Earth at a distance of around 400 pc (1300 light years), which has been extremely well studied for the past four decades. Although the source is complex, its molecular emission lines are bright enough, and the spectral and spatial resolution available to radio telescopes is high enough to allow it to be disentangled into six regions that can be differentiated in terms of density, temperature, and size (*28*). In particular, three hot, dense sources have been identified as sources of SiS emission—the Orion Hot Core (hereafter OHC), the Orion Plateau (hereafter OPl), and the Orion 15.5 km s^−1^ component (hereafter O15). The sources appear to have different heating mechanisms. The OHC does not have a central stellar source and is externally heated by the effects of nearby massive stars, while the large line widths associated with the OPl imply the presence of shocks, although the abundance of SiO detected there is more consistent with the removal of ice mantles rather than the destruction of grain cores (*28*). The heat source in the O15 source is less clear; it is defined as a clump of gas for which only SiO and SiS have peak emission at a velocity of 15.5 km s^−1^ (*28*). Silicon monosulfide has been detected in each of these clumps with observed fractional abundances relative to H_2_ of (7.1 ± 1.7) × 10^−10^ (OHC), (1.7 ± 0.4) × 10^−9^ (OPl), and (7.0 ± 1.7) × 10^−9^ (O15) (*28*). These observed values are derived on the basis of very specific choices of molecular hydrogen column densities, N(H_2_), for each source that are some of the largest inferred in interstellar clouds. Because of the great difficulty in determining N(H_2_) under these conditions—all derivations rely on either scaling up abundances of more minor components, such as isotopic carbon monoxide, or converting dust grain masses to hydrogen mass, or making approximations on source size—we conclude conservatively that the fractional abundances above are uncertain by a factor of three (Materials and Methods).

**Fig. 7:**
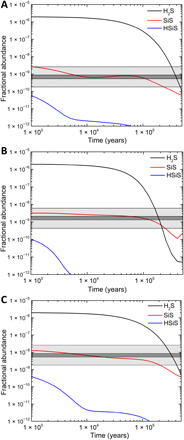
Astrochemical modeling of the Orion Hot Core. Time-dependent evolution of fractional abundances relative to molecular hydrogen of hydrogen sulfide (H_2_S), silicon monosulfide (SiS), and thiosilaformyl radical (HSiS) in models of the Orion Hot Core (**A**), the Orion Plateau (**B**), and Orion 15.5 km s^−1^ (**C**). The dark gray shaded areas show the range of silicon monosulfide (SiS) fractional abundances derived from observations, and light gray area represents the uncertainty arising from the molecular hydrogen abundance.

These models present very clear results. First, for all three star-forming regions, the fractional abundances derived for silicon monosulfide (SiS) are consistent with the astronomical observations, defined by the gray bars in Fig. 7, for chemical evolution times of 10^3^ to 10^5^ years, in agreement with the fact that these hot molecular cores are young objects with ages in the range of 10^4^ to a few 10^5^ years. Second, and not unexpectedly, the fits do not use the same underlying abundance—that in the O15 component requires a fractional abundance of atomic silicon about four times larger than the other two sources. Third, the bimolecular reaction of atomic silicon with hydrogen sulfide provides up to half of the silicon monosulfide produced, with the remaining fractional abundances accounted for by the reaction of atomic sulfur [S(^3^P)] with the silylidyne radical (SiH). Silicon and silylidyne along with sulfur are the degradation products of silane (SiH_4_) and hydrogen sulfide (H_2_S) by gas-phase binary reactions, the internal vacuum ultraviolet photon field, and energetic galactic cosmic rays (*13*, *41*, *63*). The lower fractional abundance of silicon monosulfide in the OHC is due to the fact that it is hotter and denser than the other two sources, leading to a more rapid depletion of atomic silicon at very early times, less than 1000 years, and the dominance of less efficient formation routes at longer times. Last, because our experiments reveal that silicon monosulfide (SiS) and the thiosilaformyl radical (HSiS) are formed simultaneously, the latter at the level of 5%, via the reaction of atomic silicon with hydrogen sulfide, we also calculated the fractional abundance of this species and show this in Fig. 7 with a rate coefficient for its loss with hydrogen atoms (*37*) of 10^−11^ cm^3^ s^−1^. These models predict that at a time of 10^4^ years, the thiosilaformyl radical has its largest fractional abundance, around 5 × 10^−12^, toward the O15 source with similar column densities, (5 to 8) × 10^11^ cm^−2^, in both the OHC and O15 sources. We note that if the rate coefficient for the reaction with atomic hydrogen is a factor of five larger, then the predicted thiosilaformyl column density is never more than 10^11^ cm^−2^ for times longer than about 3000 years, thus ensuring that it will be very difficult to detect.

## DISCUSSION

Our combined crossed molecular beam and electronic structure calculations provide compelling evidence on the formation of silicon monosulfide (SiS) via nonadiabatic reaction dynamics via the bimolecular neutral-neutral reaction of atomic silicon with deuterium sulfide under gas-phase single-collision conditions. In conjunction with astrochemical modeling, our study suggests that silicon monosulfide can form through this newly found reaction path toward star forming regions. This bimolecular reaction represents an important pathway to the simplest silicon-sulfur–bearing molecule—the silicon monosulfide diatomic species—as the starting point for a vigorous chemistry leading eventually to sulfide grains in deep space (*32*, *34*). Although there is, to date, no detailed description on the molecular level of how sulfide dust grains might form, silicon monosulfide is likely involved (*31*). Therefore, the work reported here represents an important step toward a systematic understanding of the fundamental mechanisms eventually leading to the formation of sulfide grains in deep space. Our studies also predict that the hitherto elusive thiosilaformyl radical (HSiS), which has a dipole moment between 3.12 (*58*) and 2.059 Debye (*64*) and whose rotational spectrum has been measured in the range of 215 to 301 GHz (*65*), may be a detectable species in hot molecular cores. Future astronomical searches for thiosilaformyl using the Atacama Large Millimeter/submillimeter Array (ALMA) would be invaluable to test this and future chemical models of the silicon-sulfur chemistry in star-forming regions and in circumstellar envelopes. An interpretation of these searches will depend on decisive developments in experimental and computational chemistry together with astrochemical modeling, thus reducing the disparity between astronomical and laboratory data on the celestial sulfur chemistry that has survived for decades and, hence, influences our assessment of organosulfur chemistry in the ISM.

## MATERIALS AND METHODS

### Experimental

The crossed molecular beam experiments of ground-state silicon atoms (Si; ^3^P) with deuterium sulfide (D_2_S; X^1^A_1_) were performed under single-collision conditions exploiting a crossed molecular beam machine (*66*). A section of the pulsed supersonic beam of neon-seeded ground-state silicon atoms was selected by a four-slit chopper defined by a peak velocity (*v*_p_) and speed ratio (*S*) of 984 ± 15 m s^−1^ and 5.9 ± 0.8, respectively. The neutral products are ionized by electron impact (Supplementary Materials), and TOF spectra were accumulated at each angle.

### Computational methods

To explore the singlet and triplet PESs of the SiSH_2_ system, geometry optimizations and vibrational frequency calculations were conducted at the CASSCF (*50*, *51*) level using the full-valence active space (12 electrons in 10 orbitals) with an aug-cc-pV(Q + d)Z (*52*) basis set. The calculations reported here were performed with the MOLPRO (*67*) package. The energies were refined using explicitly correlated multi-reference configuration interaction (MRCI-F12) calculations (*53*–*55*) exploiting the cc-pVQZ-F12 basis set (*56*). This method accurately describes bond-breaking geometries (*68*) with final values being close to the complete basis set limit. The energies also include the Davidson correction (+*Q*) to approximate quadruple excitations (*69*, *70*). The SOCs were calculated using the full spin-orbit matrix with the Breit-Pauli operator (*71*) as implemented in MOLPRO (*67*). The spin-free electronic Hamiltonian eigenstates (|*S*>, |*T*,1> |*T*,0 >, and |*T*,−1>) are used to build the total Hamiltonian matrix representation (*H*_el_ + *H*_SO_) at the MRCI level. The magnitude of the SOC (*V*_SO_) is then obtained by

$$V_{\text{SO}}^{2}=\sum_{M_{S}=-1}^{1}{|\langle T,M_{S}|H_{\text{SO}}|S\rangle |}^{2}$$

For a vibrational analysis of the MSX structures, the coordinate perpendicular to the seam must be projected out together with the rotational and translational degrees of freedom. For this, we have calculated the gradients and Hessians of the two states at the CASSCF level, which were combined to generate a new effective Hessian as described in (*72*, *73*). This effective Hessian can then be diagonalized to provide the 3*N* − 7 vibrational frequencies, which was performed using the implementation given in (*74*). These final frequencies are used for zero point energy (ZPE) corrections of the MSX energies. To correct for the neglection of anharmonic effects, all frequencies were scaled by 1.01399, which was chosen such as to improve the description of the experimental frequencies of SiS, H_2_, H_2_S, D_2_, and D_2_S.

### Astrochemical modeling

Our model calculations were carried out using the gas-phase UMIST Database for Astrochemistry (*75*). The reaction network was updated to include the reaction of silicon with hydrogen sulfide (reaction 1) together with additional neutral-neutral reactions that have been proposed recently to describe the formation of silicon and sulfur-bearing molecules including silicon monosulfide (*24*, *37*–*39*, *76*). Additional reactions necessary were included to describe the formation of the thiosilaformyl radical (HSiS) (reaction 1) and its destruction by ions such as H_3_^+^, He^+^, C^+^, and HCO^+^, by neutral species including atomic hydrogen (*37*) and by photodissociation, including those photons generated internally in the sources through cosmic-ray interactions with molecular hydrogen. The physical parameters for our three sources were taken from Tercero *et al.* (*28*), with densities in the range of 10^6^ cm^−3^ to 5 × 10^7^ cm^−3^ and temperatures from 125 to 225 K (table S4). The initial abundances adopted for the calculation of molecular evolution in hot cores usually involve one of two approaches. The first is to use a calculation of an earlier, colder phase in which accretion of species on to grain surfaces and subsequent surface chemistry to determine the initial abundances of molecules in the ice. These abundances are then adopted as the initial abundances of gas-phase species on the assumption that the ice mantles are sublimed upon heating by a nearby star. The second neglects the physical and chemical evolution that goes on in the earlier phase to adopt initial gas-phase abundances that are consistent with the molecular abundances that are actually observed in interstellar ices. Both approaches have their limitations—the first in that abundances are very sensitive to the physical cloud model adopted, the time dependence of density and temperature for example, and to the grain properties such as composition, porosity, and temperature. Diffusion rates on surfaces are exponentially dependent on the ratio of binding energies to grain temperature, neither of which is well known. In the second approach, species with surface abundances less than about 1% of water are undetectable so that only a limited set of abundances can be defined, and the abundances of these few species are known to vary from source to source. Here, we have adopted the second approach, and table S5 lists the initial abundances used in our model calculations. Because of the low abundances of sulfur and silicon relative to carbon, nitrogen, and oxygen, our results are not very dependent on the initial abundances of species containing these latter elements.

At the temperatures of our three sources, the chemistry is initiated by ice sublimation (*62*), and we adopt an initial fractional abundance of hydrogen sulfide of 2 × 10^−6^, similar to the value (1.2–4.2) × 10^−6^ derived by Crockett *et al.* (*77*) toward the OHC; this accounts for about 10% of the elemental sulfur (*76*). Although the cosmic abundance of silicon is roughly a factor of two larger than that of sulfur, it is established that a very large percentage of elemental silicon is locked (or depleted) within refractory grain cores and cannot be released to the gas unless interstellar shocks destroy the grain cores. For this reason, the fractional abundance of silicon that is contained in interstellar ices is less than that of sulfur. Here, we adopt an initial abundance of silane (SiH_4_) of 2 × 10^−7^ and an atomic silicon abundance of 8 × 10^−9^.

## SUPPLEMENTARY MATERIALS

Supplementary material for this article is available at http://advances.sciencemag.org/cgi/content/full/7/26/eabg7003/DC1
